# The effectiveness and influencing factors of the “Y” line technique in reducing the leg length discrepancy after total hip arthroplasty

**DOI:** 10.3389/fsurg.2023.1074103

**Published:** 2023-03-06

**Authors:** Wenshu Jin, Huaqiang Sun, Xudong Duan, Yange Gu, Zhang Zhao, Xinfeng Yan

**Affiliations:** ^1^ School of Sports Medicine and Rehabilitation, Shandong First Medical University and Shandong Academy of Medical Sciences, Tai an, China; ^2^ Department of Orthopedic Surgery, The First Affiliated Hospital of Shandong First Medical University and Shandong Provincial Qianfoshan Hospital, Shandong Key Laboratory of Rheumatic Disease and Translational Medicine, Jinan, China; ^3^ Cheeloo College of Medicine, Shandong University, Jinan, China

**Keywords:** total hip arthroplasty, THA, leg length discrepancy, LLD, surgical techniques

## Abstract

**Objective:**

To introduce a surgical technique (the “Y” line technique) that will control leg length discrepancy (LLD) after total hip arthroplasty and to observe its effectiveness and influencing factors.

**Methods:**

According to the inclusion and exclusion criteria, a total of 350 patients were selected in this study; 134 patients in whom used the “Y” line technique was used to control lower limb length were included in Group A and 166 patients treated with freehand methods to control lower limb length were included in Group B. A total of 50 patients in whom the standard anteroposterior x-ray of bilateral hips was taken preoperatively and in whom the “Y” line technique was used during the operation were included in Group C.

**Results:**

The postoperative LLD of Group A was 4.74 mm (3.93), that of Group B was 5.85 mm (4.60), and that of Group C was 2 mm (1.00)—the difference was statistically significant (*p* < 0.001). There were significant statistical differences when comparisons were made between any two groups (*p* < 0.01). The distribution of postoperative LLD in Group A was better than that in Group B, and this factor was better in Group C than in Group A—the difference was statistically significant (*p* < 0.001). Severe unequal length rates of the lower extremities (LLD > 10 mm) were 5.97% (8/134) in Group A, 14.3% (24/166) in Group B, and 0% (0/50) in Group C—the difference was statistically significant (*p *< 0.001). There were significant differences between Group A and Group B and between Group B and Group C (*p* < 0.05), but there was no significant difference between Group A and Group C (*p* = 0.078).

**Conclusion:**

The “Y” line technique, which does not increase the operating time and patient cost, can effectively reduce postoperative LLD. Insufficient internal rotation of the healthy lower extremity and the low projection position in the preoperative anteroposterior x-ray of the bilateral hips were important factors affecting the accuracy of the “Y” line technique.

## 1. Introduction

Leg length discrepancy (LLD) is a common complication that occurs after total hip arthroplasty (THA), and it is also the main reason why patients are dissatisfied with the operation (1). Severe LLD can lead to gait disorders, lower back pain, hip dislocation, sciatica, prosthesis loosening, and even early revision problems (2–5). At present, there are many methods to control LLD (6, 7), but most of them have disadvantages such as cumbersome use, the need for additional equipment, increased operating time or cost, low accuracy, and so on.

This paper introduced a new method (the “Y” line technique) to control LLD by measuring the central height of the acetabulum and femoral head of the healthy hip on preoperative x-ray and adjusting the prosthesis height according to the preoperative measurement intraoperatively. Good results were obtained, and the factors affecting the accuracy of this method were also analyzed.

## 2. Materials and methods

### 2.1. General information

The Ethics Committee of the First Affiliated Hospital of Shandong First Medical University (IRB No. 2021-S943) approved this single-center retrospective study and waived the need for written informed consent for participation in the study. The inclusion criteria were (1) unilateral hip abnormality with a normal contralateral hip; (2) no obvious scoliosis or pelvic tilt. The exclusion criteria were (1) intraoperative femoral osteotomy; (2) appreciable dysplasia of the pelvis and lower limbs; the top of the greater and lesser trochanters, or the teardrop were clearly unidentifiable on plain radiographs.

According to the inclusion and exclusion criteria, a total of 350 patients from the First Affiliated Hospital of Shandong First Medical University were selected for this study in the period between June 2017 and July 2020. In Group A (134 cases) patients, the “Y” line technique was used to control the length of the lower limbs. Group B (166 cases) patients were treated by freehand methods to control lower limb length. An additional 50 patients constituted Group C, who were subjected to a standard anteroposterior x-ray of the bilateral hips for preoperative measurement. The standard photographic method used was the supine technique, with the hips (at least the healthy hip) fully extended and internally rotated by 15°–20°, the projection point kept straight above the midpoint of the bilateral hips, and the projection distance kept at 1 m. The length of the lower limbs in Group C patients was controlled by the “Y” line technique during the performance of the operation. The general data are presented in Table 1.

**Table 1:** General information of patients.

	Group A	Group B	Group C
The number of cases	134	166	50
Gender (M/F)	69/65	85/81	20/30
Age (years)	56.95 ± 10.51	61.57 ± 11.66	59.38 ± 9.602
ONFH^a^	66	78	21
Osteoarthritis^b^	47	56	19
Femoral neck fracture	21	32	10
BMI (kg/m^2^)	25.01 ± 3.34	26.09 ± 3.60	25.41 ± 3.27

^a^ Osteonecrosis of femoral head.

^b^ Degree I developmental dysplaisa of the hip were included.

### 2.2. Research methods

During the performance of all the surgical procedures, the patients were placed in the lateral position, and the posterolateral approach was used. Three kinds of femoral stems that were mostly similar in shape were used in this study, so as to make the study more comparable between the groups. In Group A and Group C patients, a cementless prostheses BE femoral stem (Beijing Chunlizhengda Medical Instruments Co.) was used, and the “Y” line was drawn on its femoral rasp holder that was located at the top of the femoral head center. In Group B patients, two kinds of cementless prostheses were used, which were 60 CL femoral stems (AK Medical Holding Limited) and 106 Corail femoral stems (Johnson & Johnson/DePuy). No “Y” line was drawn on the CL and Corail femoral rasp holder, but during the performance of the procedures, the surgeons also followed the principles of the “Y” line technique by way of visual inspection and other freehand methods to control LLD.

The method of controlling the leg length discrepancy by the “Y” line technique is as follows. The basic principle of the “Y” line technique is measuring the distance from the center of the acetabulum to the line that connects the two teardrops (the height of the acetabulum) and the distance from the center of the femoral head to the greater trochanter plane (the height of the femoral head) of the healthy hip on the preoperative x-ray film. During the operation, after installing the prosthesis, an attempt was made to make the height of the acetabular prosthesis center and the height of the femoral head prosthesis center align with that of the healthy hip, or correspondingly, to move the two centers upwards/downwards, so as to achieve the same length of the bilateral lower limbs.

### 2.3. Preoperative measurement of the healthy hip on the preoperative x-ray film

#### 2.3.1. Measurement of the height of the acetabulum A

First, the H-line was drawn—the line through the lowest point of the two teardrops (the lower edge of the bony acetabulum)—and the rotation center (spot O) of the femoral head (it was also the center of the acetabulum) was found. Then, the distance from O to the H-line was measured, which was the acetabulum height A.

#### 2.3.2. Measurement of the height of the femoral head B

**Figure 1:**
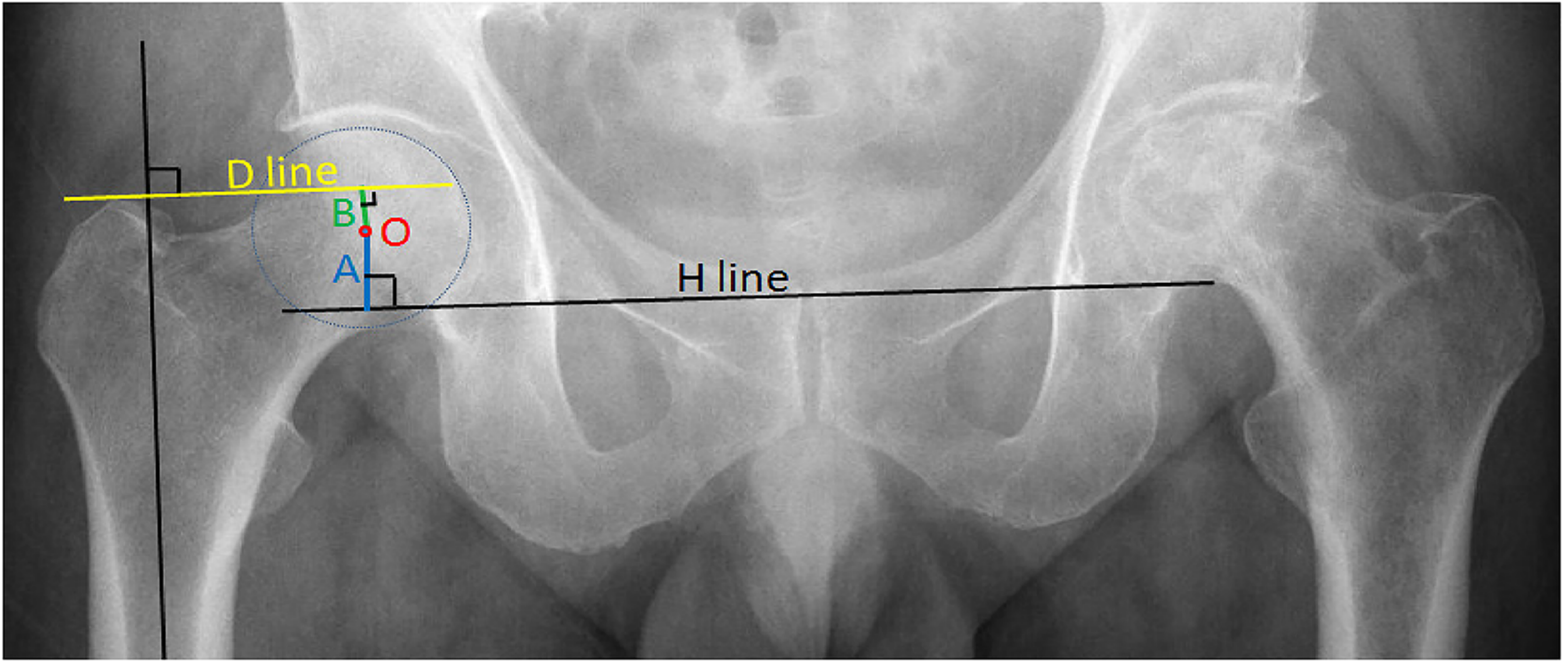
Preoperative measurement of a healthy hip: A is the height of the acetabulum, and B is the height of the femoral head.

Second, the D-line was drawn through the upper end of the great trochanter and vertical to the femoral longitudinal axis. Then, the distance from O to the D-line was measured, which was defined as the height of the femoral head B (if point O was above the D-line, a positive value was recorded; if point O was under the D-line, a negative value was recorded) (Figure 1).

### 2.4. Intraoperative measurement

#### 2.4.1. Measurement of the height of the acetabular cup A'

**Figure 2:**
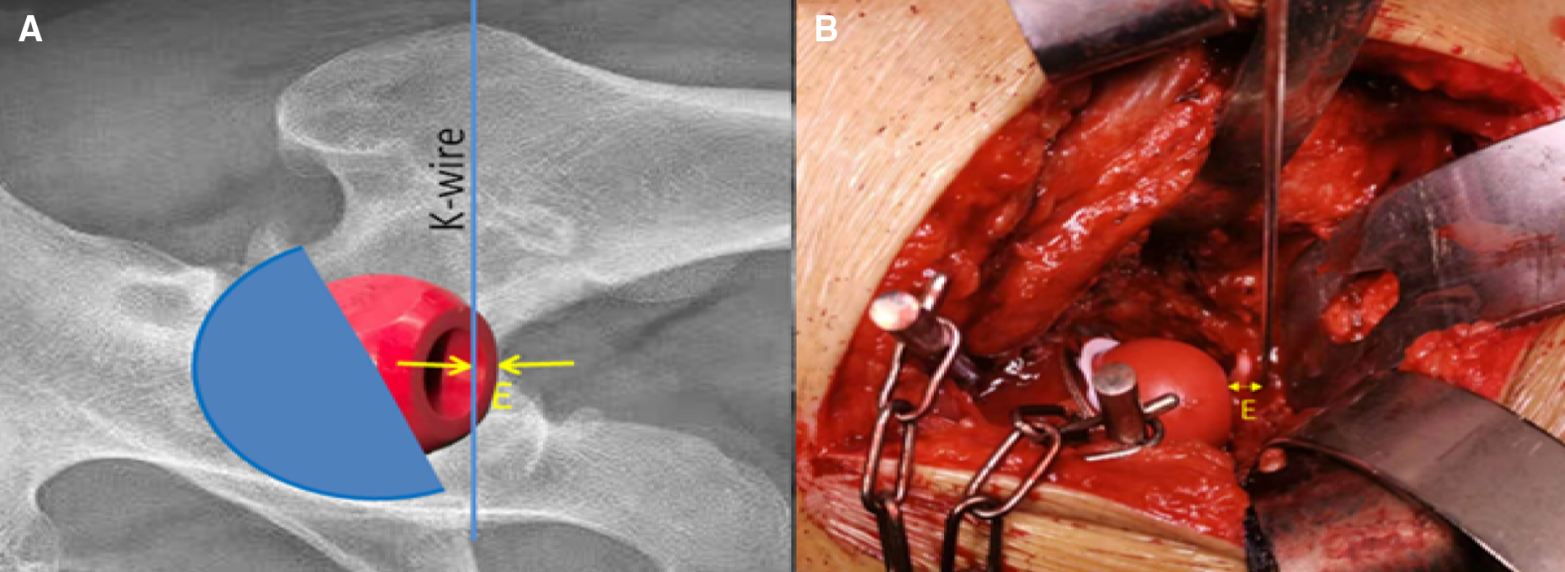
Intraoperative measurement of the height of the acetabular cup: A': A' = r + E for the right picture, and A' = r − E for the left picture.

After implanting the acetabular cup and liner, a femoral head trial was placed into the liner. Then, a Kirschner wire was placed perpendicularly to the operating table, and if necessary, close to the inferior margin of the bone acetabulum (that is the lower edge of the teardrop on the x-ray film), and the osteophytes that covered the inferior margin of the bone acetabulum by chisel were removed. The height of the acetabulum cup A' = the radius of the femoral head trial (r) + the distance from the femoral head trial to the Kirschner wire (E). If the Kirschner wire was further from the farthest point of the femoral head trial, E was recorded as a positive value (see Figure 2B). Otherwise, the defective part of the femoral head trial was used to face the Kirschner wire to measure the E value and record a negative value (see Figure 2A).

#### 2.4.2. Measurement of the height of the femoral head B'

**Figure 3:**
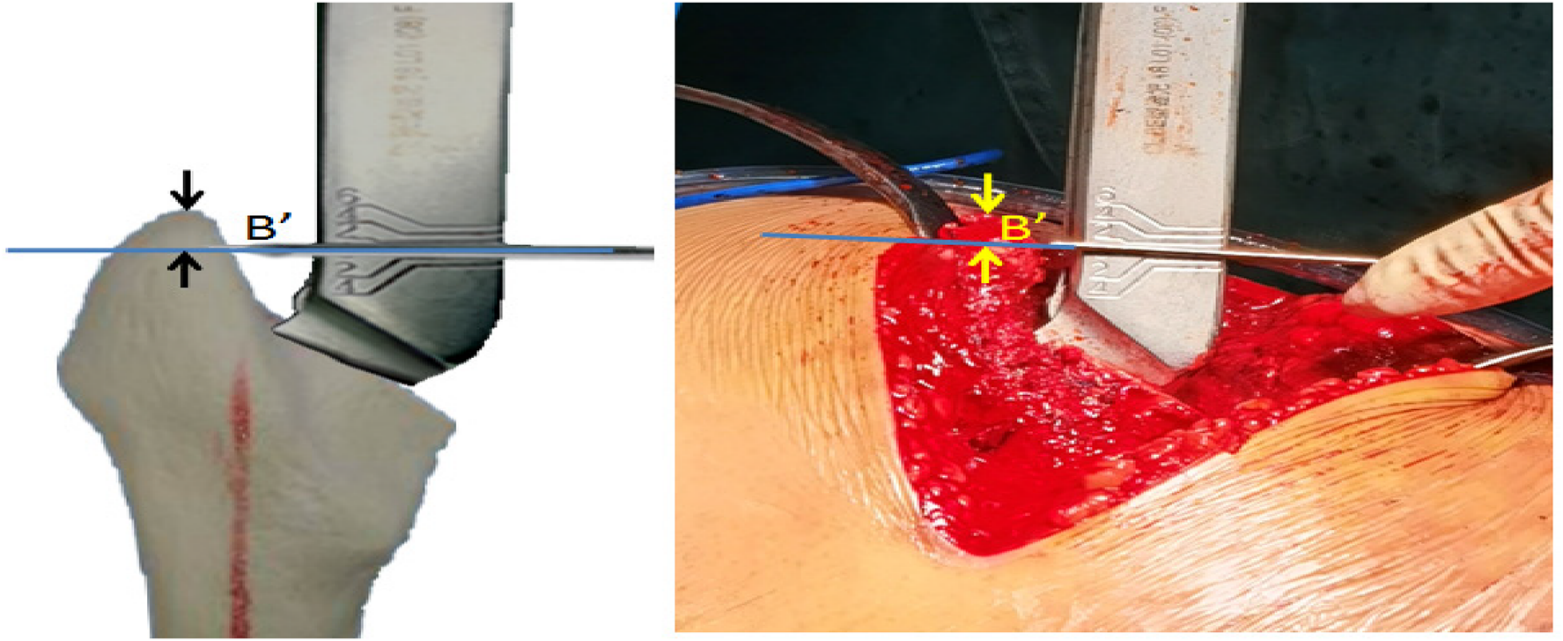
Intraoperative measurement of the height of femoral head B'.

The “Y” line is one of a group of horizontal lines perpendicular to the longitudinal axis of the femoral stem rasp on the rasp holder, which was marked with “0” and was exactly located at the height of the femoral head center when installing the standard length of the femoral head. After the optimal rasp was placed, the height of the femoral head B' could be obtained by measuring the distance from the top of the greater trochanter (paying attention to the soft tissue) to the “Y” line by using a Kirschner wire to extend the line to the great trochanter. If the line was above the greater trochanter, B' was recorded as a positive value; otherwise, B' was recorded as a negative value (see Figure 3).

#### 2.4.3. Adjusting the height of the femoral head during the operation

To make the two leg lengths equal after surgery, the formula A − A' = B − B' should be used. This formula states that the height of the acetabular cup should be moved upwards or downwards compared with that of the healthy hip, and the femoral head height should be moved upwards or downwards at the same distance accordingly. Since the A' value was fixed once the acetabular prosthesis was installed, it was necessary to adjust the height of the femoral head to the optimal B' value by using different sizes of stems and/or different lengths of femoral heads to meet A − A' = B − B'.

### 2.5. Control method in Group B patients during operation

All surgeons were familiar with the principle of the “Y” line technology to be applied in Group B patients. But there was no “Y” line on their rasp holders; thus, they only visually used this principle during the operation as well as other freehand LLD controlling methods such as palpating the two knees, Shuck Test, palpating iliotibial tract tension, and so on.

### 2.6. Postoperative measurement

On the postoperative anteroposterior radiographs of the bilateral hips, the distances from the top of the bilateral lesser trochanters to the H-line were measured; the difference of the two distances was considered the LLD value. The LLD was set to be positive when the affected limb was longer than the healthy limb and negative if otherwise.

### 2.7. Statistical methods

All statistical analyses were performed by using SPSS software for Windows (version 25.0 SPSS, New York, United States), and a score of *p* < 0.05 was considered statistically significant. All sample data of the three groups (Groups A, B, and C) were of non-normal distribution. The Kruskal–Wallis test of measurement data, Chi-square test of ratios, and the Chi-square test of grade data were used to compare the differences between postoperative LLD among the three groups.

## 3. Results

**Figure 4:**
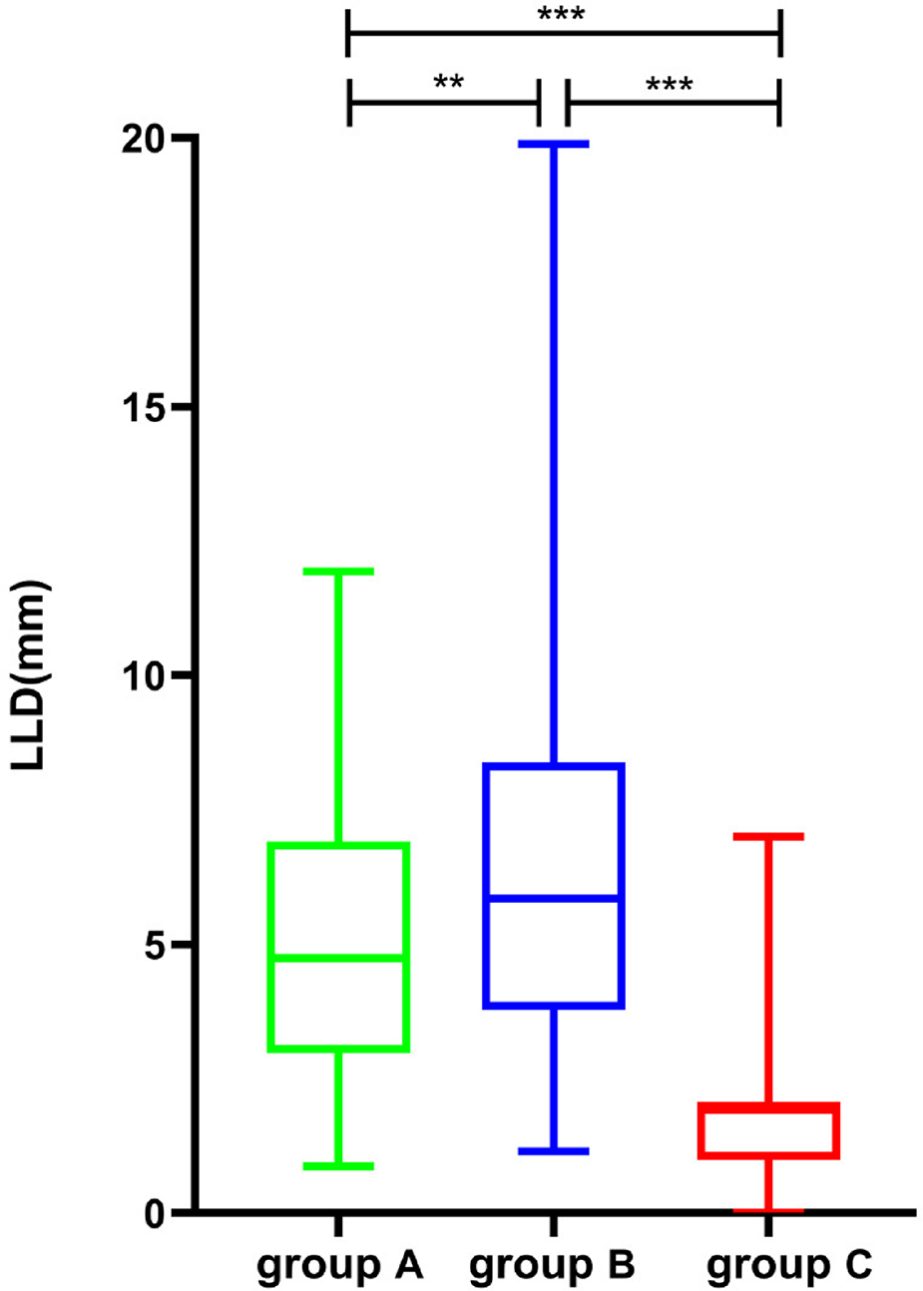
Comparison of postoperative LLD in patients of groups A, B, and C (**p < 0.01, ***p < 0.001). LLD, leg length discrepancy.

**Figure 5:**
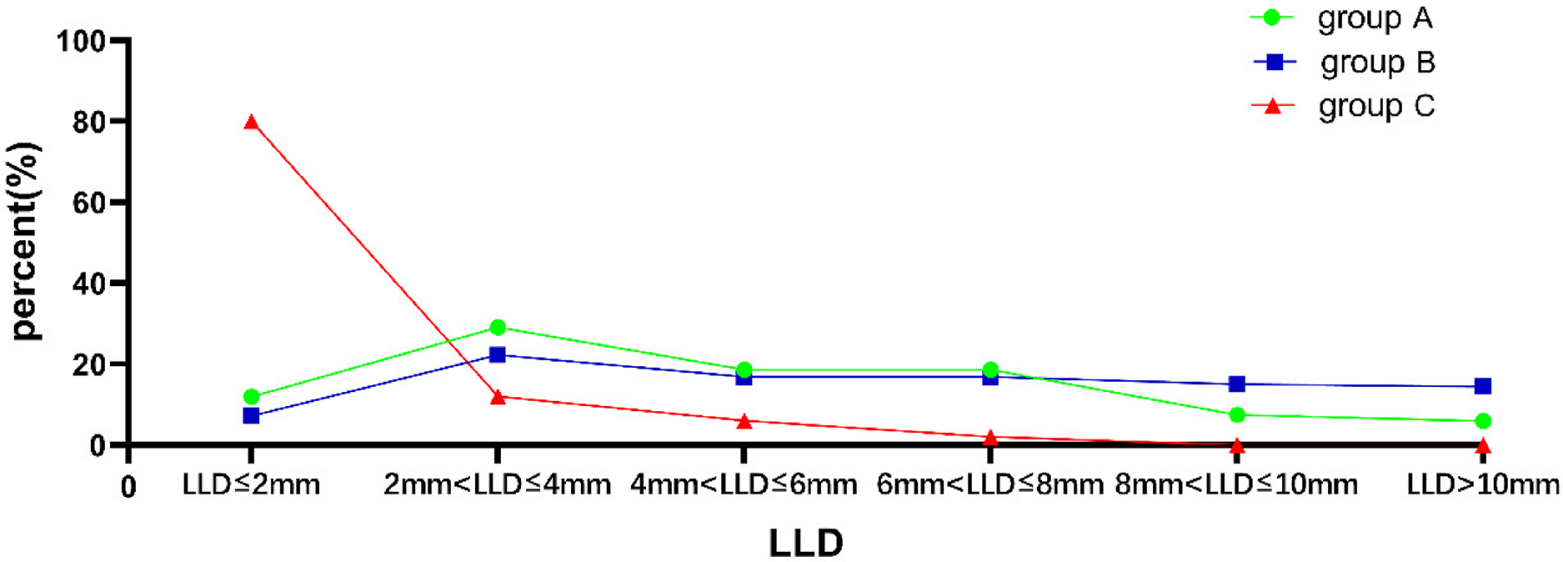
Comparison of the distribution of postoperative LLD in patients of groups A, B, and C (*p* < 0.001). LLD, leg length discrepancy.

**Figure 6:**
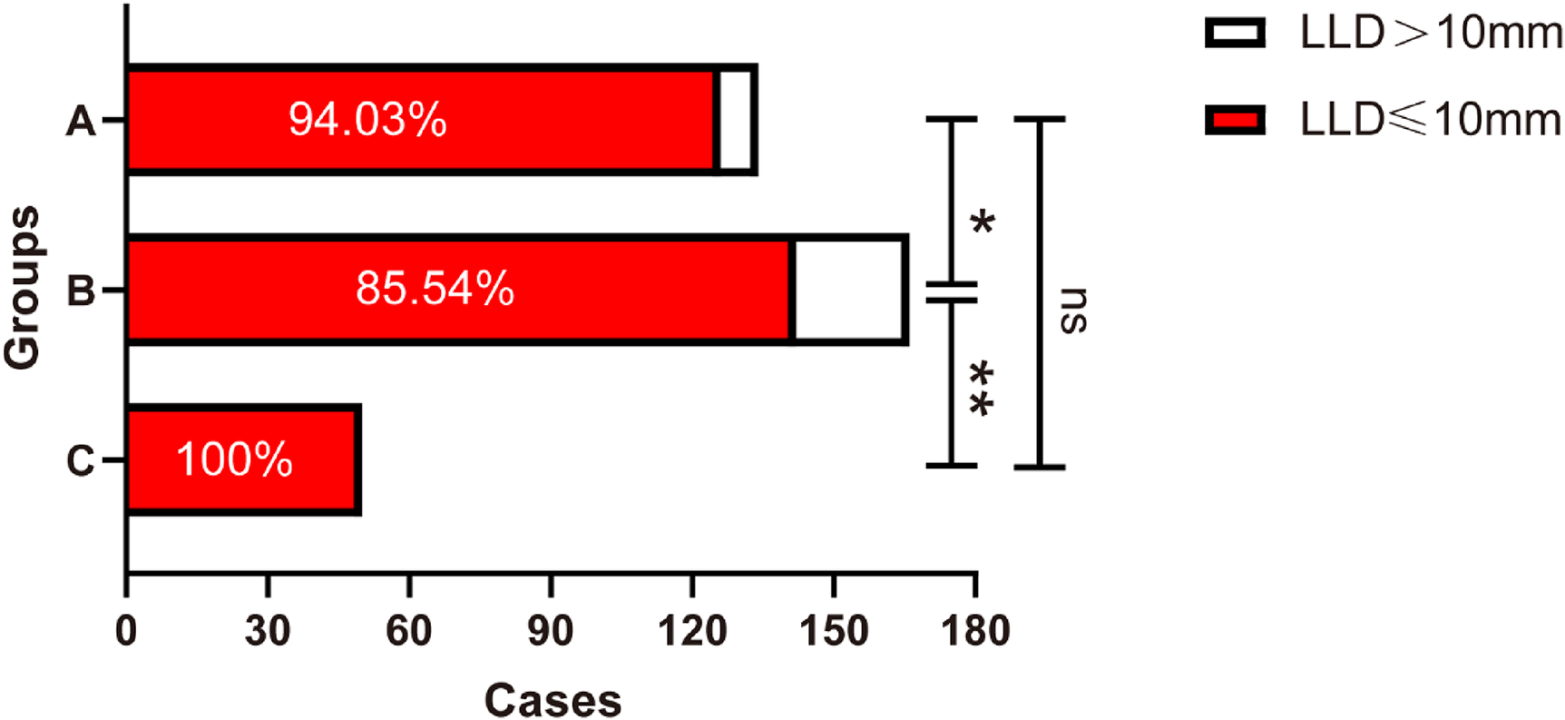
Comparison of the proportion of postoperative LLD > 10 mm in patients of groups A, B, and C (*p < 0.05, **p < 0.01; ns indicates no significance). LLD, leg length discrepancy.

1. Average postoperative LLD was 4.74 mm (3.93) in Group A, 5.85 mm (4.60) in Group B, and 2 mm (1.00) in Group C. The Kruskal–Wallis test was used to compare the postoperative LLD of the three groups, and the difference was statistically significant (*Z* = 86.689, *P *< 0.001). There were significant differences between Group A and Group B (*P* < 0.002), Group B and Group C (*P* < 0.001), and Group A and Group C (*P* < 0.001, Figure 4).
2. The distribution of postoperative LLD in the three groups is shown in Table 2. In Group C, the longest LLD was only 7 mm. The Chi-square test was used to compare the postoperative LLD distribution among the three groups, and the difference was statistically significant (*χ*² = 89.263, *p *< 0.001). The LLD in Group C was significantly smaller than that in Group A (*p* < 0.001) and Group B (*p* < 0.001), and the LLD in Group A was smaller than that in Group B (*p* = 0.002, Figure 5).
3. The proportion of patients with postoperative LLD greater than 10 mm was 5.97% (8/134) in Group A, 14.3% (24/166) in Group B, and 0 in Group C. The Chi-square test was used and the difference was statistically significant (*χ*² = 12.265, *p *= 0.002). There were significant differences between Group A and Group B (*p* = 0.018) and between Group B and Group C (*p* = 0.004), but there was no significant difference between Group A and Group C (*p* = 0.078, Figure 6).

**Table 2:** The distribution of postoperative LLD in groups A, B, and C.

	The distribution of postoperative LLD					
Groups	≤2 mm	2–4 mm	4–6 mm	6–8 mm	8–10 mm	>10 mm
A (cases)	16	39	36	25	10	8
B (cases)	12	37	40	28	25	24
C (cases)	40	6	3	1	0	0

LLD, leg length discrepancy.

## 4. Discussion

### 4.1. The effect of unequal lower limb length after total hip arthroplasty

LLD can affect the daily life of patients in varying degrees and significantly reduce the patient's postoperative quality of life (2–5). With the development of THA surgery technology, LLD has seen a significant decrease but not complete elimination (6–8). With regard to the range of LLD that patients can tolerate, no definite conclusion has been reached yet. Maloney and Keeney (9) opined that no symptoms will occur when LLD was less than 10 mm after total hip arthroplasty; however, some patients found it difficult to tolerate even a very small range of LLD (10). In this study, the percentage of postoperative LLD > 10 mm and the distribution of LLD were statistically compared, and the results showed that the complete and separate use of the “Y” line technique could help control LLD better than when this technique is visually combined with other freehand methods.

### 4.2. Advantages of “Y” line technology

The most commonly used freehand methods to reduce LLD after THA are palpating the two knees, palpating iliotibial tract tension, and the Shuck Test. However, such methods yield inaccurate results because of the influence of body position and the types of anesthesia used (11). An intraoperative device (12), navigation system (13, 14), and intraoperative fluoroscopy (15–18) could help reduce LLD, but these systems and processes require the use of more surgical equipment or procedures, which will increase the cost and/or operating time and also the risk of infection. The control of LLD by solely relying on the preoperative measurement of the template was still unreliable (19) in this study, but if this was combined with the intraoperative measurement of the height of the femoral head prosthesis, LLD could be more effectively reduced (20). However, the study did not consider the height of the acetabulum, and some studies found that the height of the acetabulum changed in varying degrees after THA compared with that before the operation (21).

The “Y” line technique takes into account the preoperative measurement, the intraoperative changes in the height of the acetabulum, and the height of the femoral head simultaneously, all of which indicate that it is theoretically more accurate.

In this study, the surgeons used the “Y” line technical principle without the “Y” line rasp holder in Group B patients, but they did not obtain good results as in Group A and Group C patients, in whom they used the femoral rasp holder with the “Y” line drawn on it. This further confirms the reliability of the complete and separate use of “Y” line technology in reducing LLD. In addition, by comparing the distribution of postoperative LLD between Group A and Group C patients, it was found that taking a standard preoperative anteroposterior x-ray of the bilateral hips and using the “Y” line technique were more effective in reducing LLD than other approaches.

“Y” line technology uses only routine operative instruments, does not increase the number of surgical steps, and does not require additional measuring tools or equipment; therefore, it provides ease of use without any extra cost. “Y” line technology was little affected by the patient position. If the preoperative anteroposterior x-ray of the bilateral hips is taken according to the standard procedure, this method can achieve high accuracy without increased operating time and cost. The results of this study suggest that, not only does the incidence of postoperative LLD > 10 mm decrease significantly, but also the average postoperative LLD decreases significantly in patients in whom this method was used.

### 4.3. Factors affecting the use of “Y” line technology

The quality of the preoperative anteroposterior x-ray of the bilateral hips will influence the measured value (22) and has the greatest impact on the use of “Y” line technology. The results of the x-rays on Group C patients showed that the standard anteroposterior x-ray of the bilateral hips could greatly reduce LLD, but at the same time suggest that some x-rays provided by the imaging department may be substandard.

**Figure 7:**
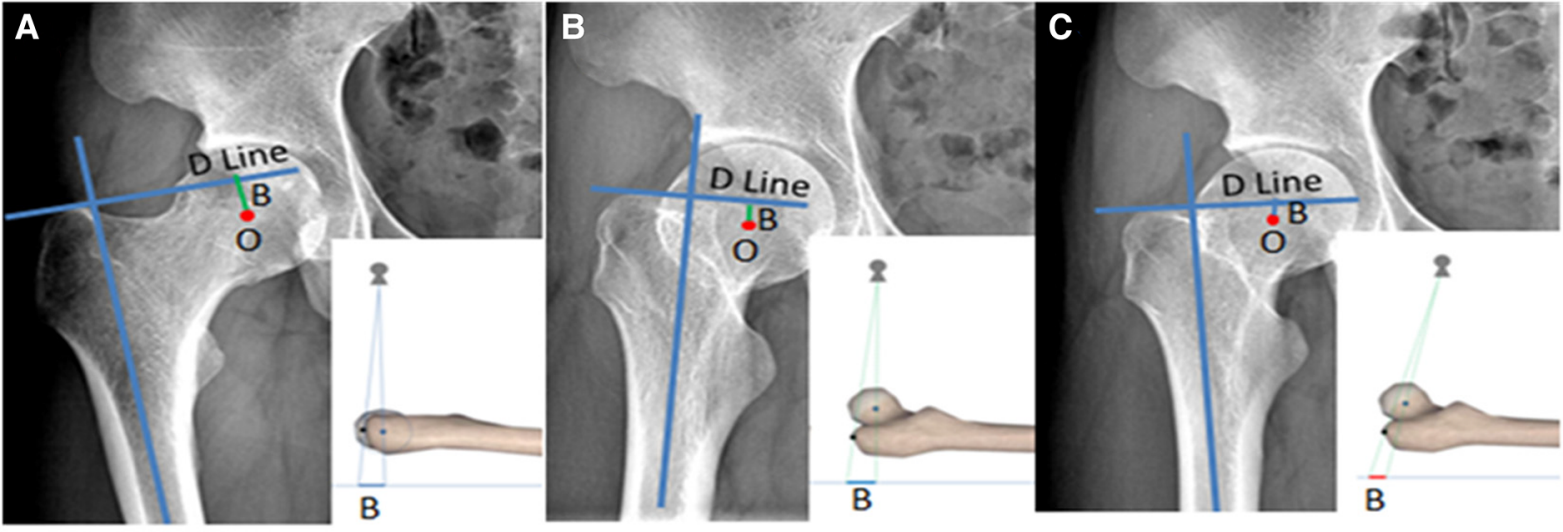
Bilateral hip x-ray of the same volunteer: (**A**) is the standard photographic method as previously mentioned, (**B**) is the externally rotated hip and the projection point is standard, and (**C**) is the externally rotated hip and the projection point is 10 cm below the midpoint of the bilateral hips; the femoral head center is significantly higher than the actual value shown in (**A**).

If the lower limbs are not sufficiently externally rotated, the center point of the femoral head and the apex of the greater trochanter will not be on the same plane due to the anteversion angle of the femoral neck. When this happens along with a very low projection point, the measured height of the femoral head will be significantly higher than the actual value, leading to postoperative extremity lengthening, as shown in Figure 7. The condition of five among eight patients with postoperative LLD > 10 mm in Group A was related to the substandard preoperative x-rays as described above.

It can be discerned from the above observation that hip external rotation and a low projection point will significantly influence the use of “Y” line technology (Figure 7). To avoid this influencing factor, when the lesser trochanter is found to be too large and the femoral calcar is not displayed clearly, or Shenton’s line is discontinuous on the preoperative x-ray, it is necessary to take an x-ray again under standard conditions, because these phenomena indicate that the internal rotation of the hip is insufficient and the projection point is dislocated.

## 5. Conclusion

In total hip arthroplasty, the use of the femoral rasp holder with a “Y” line to implement a complete “Y” line technique can help control postoperative LLD more effectively than the visual “Y” line technique combined with a comprehensive freehand method. Insufficient internal rotation of the hip and low projection position when taking a preoperative anteroposterior x-ray of the bilateral hips are important factors affecting the accuracy of the “Y” line technique.

## Data Availability

The original contributions presented in the study are included in the article/Supplementary Material, further inquiries can be directed to the corresponding authors.
